# Shunt revision requirements after posthemorrhagic hydrocephalus of prematurity: insight into the time course of shunt dependency

**DOI:** 10.1186/2045-8118-12-S1-O22

**Published:** 2015-09-18

**Authors:** Edward Ahn, Joanna Wang, Eric Jackson, George Jallo

**Affiliations:** 1 Johns Hopkins University School of Medicine, USA

## Introduction

Intraventricular hemorrhage (IVH) is a common affliction of preterm infants, and often results in posthemorrhagic hydrocephalus (PHH). These patients typically eventually require permanent CSF diversion and are presumed to be indefinitely shunt-dependent. To date, however, there has been no study of long-term shunt revision requirements in patients with PHH.

## Methods

We analyzed retrospectively collected data for 89 preterm patients diagnosed with Grade III and IV IVH and PHH at our institution from 1998 to 2011.

## Results

69 out of 89 patients (77.5%) underwent VP shunt placement, and 33 (47.8%) required at least one shunt revision and 18 (26.1%) required multiple revisions. The mean ± standard deviation follow-up time for shunted patients was 5.0 ± 3.3 years. The majority of early failures are due to proximal catheter malfunction, while later failures were mostly due to distal catheter problems. There was a significant difference in the number of patients requiring revisions in the first three years following initial VP shunt insertion compared after three years, with 28 revisions versus 10 (p<0.004). In the 10 patients who underwent shunt revisions after three years, evidence of obstructive hydrocephalus was found on imaging either in the form of an isolated fourth ventricular cyst or aqueductal stenosis.

## Conclusions

Our results suggest that in a distinct subset of patients with PHH, obstructive hydrocephalus may develop, resulting in long-term dependence on CSF diversion. Further study on the factors associated with long-term shunt dependence and revision requirements within the PHH group is warranted.
